# Split red blood cell units contain defined extracellular K^+^ levels, which are improved by a washing procedure

**DOI:** 10.1111/vox.70004

**Published:** 2025-02-17

**Authors:** Fabiola Hoppe, Jacqueline Maier, Holger Kirsten, Martin Federbusch, Reinhard Henschler

**Affiliations:** ^1^ Institute of Transfusion Medicine, Faculty of Medicine Leipzig University Leipzig Germany; ^2^ Institute for Medical Informatics, Statistics and Epidemiology (IMISE), Faculty of Medicine Leipzig University Leipzig Germany; ^3^ Institute of Laboratory Medicine, Faculty of Medicine Leipzig University Leipzig Germany

**Keywords:** hyperkalaemia, infants, potassium, red blood cell, transfusion, washing

## Abstract

**Background and Objectives:**

We should control free K^+^ during massive transfusion (>80 mL/kg) of red blood cells (RBCs) in small children. To manage this, several national and international guidelines recommend using RBCs stored only up to 7 days. We tested a washing step for RBCs in saline‐adenine‐glucose‐mannitol (SAGM) with or without irradiation to reduce supernatant K^+^ levels, improve quality and potentially extend the shelf life of stored RBCs.

**Materials and Methods:**

RBCs of 240–330 mL were prepared from whole blood donations, then split into halves and stored in SAGM solution at 4 ± 2°C for 21 days. RBCs were split and washed on Days 1 and 8, and some were gamma‐irradiated on Day 8. Glucose, lactate, lactate dehydrogenase (LDH), adenosine triphosphate (ATP), K^+^ and haemolysis were determined over 21 days.

**Results:**

After washing on Day 1, only glucose and lactate improved, whereas after washing on Day 8, LDH and K^+^ also improved. Irradiation resulted in accelerated K^+^ accumulation and increased haemolysis. Mean extracellular K^+^ concentrations were 21.2 ± 1.03 mM after irradiation on Day 8 versus 1.12 ± 0.05 mM after irradiation plus wash on Day 8, and 38.80 ± 2.13 mM on Day 10 after irradiation on Day 8 and 16.6 ± 0.05 mM on Day 10 after irradiation plus wash on Day 8.

**Conclusion:**

K^+^ concentrations remained <25 mM within 8 days of storage. We recommend irradiation by Day 8 at the latest for neonatal transfusion. The shelf life may be extended by another 48 h if the RBCs are also washed.

**Highlights**

- K^+^ concentrations in red blood cell (RBC) units stored in saline‐adenine‐glucose‐mannitol reached levels of up to 25 mM on Day 8.
- Adenosine triphosphate and haemolysis were affected by washing or, to a lesser extent, by irradiation.
- A washing step is useful to lower K^+^ levels in RBCs for neonatal massive transfusion; it extends shelf life by a further 2 days.

## INTRODUCTION

The quality of red blood cell (RBC) product has received particular attention in perinatal transfusion medicine because K^+^ accumulates in the plasma of stored RBC, and this accumulation is transfused with the RBC [1, 2, 3, 4]. Whereas peri‐ and neonatal transfusions are normally of modest RBC volumes (≤20 mL/kg over 3–4 h or ≤5 mL/kg/h), massive transfusions of >80 mL/kg in 24 h, corresponding to ≥25 mL/kg or >5 mL/kg/h, can cause transfusion‐associated hyperkalaemic cardiac arrest (TAHCA) and death [3, 4]. Massive paediatric transfusion may include intrauterine transfusions, filling of extracorporeal circulation (EC) machinery, for example, for cardiopulmonary bypass operations, and exchange transfusions. The latter involve double blood volume exchanges for which 160–200 mL/kg are needed to remove around 90% of red cells and 50% of bilirubin [4, 5]. One study did not demonstrate an impact of RBCs stored for periods of 7 days or less in comparison to units stored for an average of 14 days [6], although it was not specifically focused on massive transfusion in neonates. Recent literature suggests that in paediatric massive RBC transfusions, factors such as rapid transfusion rates, rather than the total volume transfused, as well as low cardiac output and the age of the blood component, may serve as confounders in the development of TAHCA [1, 4]. In addition to co‐transfused K^+^, contact of artificial surfaces with red cells, the use of central lines and kidney dysfunction also have been associated with increased haemolysis and hyperkalaemia [1, 4].

National and international guidelines currently recommend different storage limits of paediatric RBCs between 5 and 7 days and that transfusion should be performed within 24 h after irradiation for peri‐ and neonatal transfusions [7, 8, 9, 10]. Washing of RBCs prior to transfusion has been suggested to reduce extracellular K^+^ in paediatric components [11, 12, 13, 14]. Still, harmonized recommendations are missing, and there is no consensus on minimal quality parameters for RBCs that can be issued for paediatric massive transfusion. Units may be split, either into halves or quarters. We aimed to find out whether a wash during early storage of split red cell concentrates would improve the quality of the component for neonatal transfusions, specifically in terms of supernatant potassium levels, and whether it may allow shelf life extension.

## MATERIALS AND METHODS

Routine whole blood donations of 450 mL from voluntary donors were collected into citrate phosphate dextrose top‐and‐bottom type collection bags (LQT6281FC, MacoPharma, Mouvaux, France). After an overnight hold of 12–18 h at 20–22°C, bags were centrifuged at 5000 *g* for 14 min and separated by a Macopress Smart EVO automated blood component extractor (Macopharma) using the buffy coat method. After leucodepletion through an LCRD Leucoflex filter, RBCs were suspended in 100‐mL saline‐adenine‐glucose‐mannitol (SAGM) and stored at 2–6°C overnight. RBCs were split into two equal parts using a sterile tubing welder (TSCD‐II, Terumo BCT, USA) into collection bags (X6R 2247, Fresenius KABI, Germany). RBCs were washed twice in SAGM (Macopharma, Mouvaux, France) according to the established standard process by centrifugation in a Roto Silenta 63/630 RS‐centrifuge (Hettich, Tuttlingen, Germany) at 2489 *g* for 14 min at 20 ± 2°C. After centrifugation, the supernatants of the treatment group were removed via a manual plasma extractor (Fenwal Laboratories, Deerfield, USA), and RBCs were resuspended in SAGM up to the weight of the pre‐wash RBCs.

**FIGURE 1:**
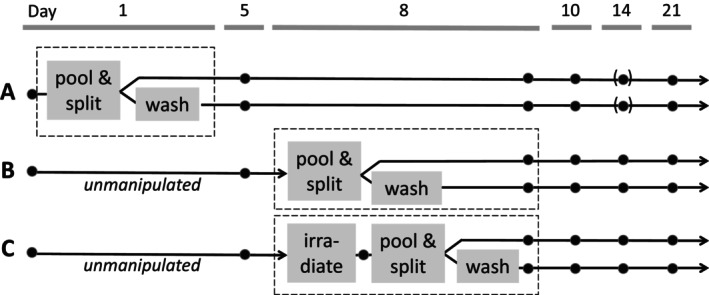
Experimental layout. The study Series A, B, and C, time points of the pooling and splitting, washing and irradiation procedures are shown. *N* was 4 (Series A), 11 (Series B) and 11 (Series C). Brackets indicate values that were planned but not obtained. The day of collection was Day 0.

To assess best and worst‐case scenarios, three series of experiments (A–C) were performed (Figure 1). RBCs were split into two equal portions either on Day 1 post‐collection (Series A; *n* = 4) or on Day 8 post‐collection (Series B and C; *n* = 11 each). The volume range of the split units was 120–165 mL. One component of each pair was washed by centrifugation and SAGM solution added afterwards. In Experiment C, RBCs (*n* = 22; 11 per arm) were in addition gamma‐irradiated with 30 Gy using a ^137^Cs source (Gamma‐Service Medical GmbH, Germany, Leipzig) on Day 8 and subsequently washed (treatment group) or not (controls). In the following, ‘unmanipulated’ refers to the original RBCs in Series B and C before they were split, whereas ‘unwashed’ defines control samples not undergoing the washing procedure (Figure 1).

Red cell indices including haemoglobin (Hb), hematocrit and mean corpuscular volume were analysed using a CELL‐DYN Ruby haematology analyser (Abbott Diagnostics, Illinois, USA). Lactate, extracellular K^+^, free Hb, haemolysis, lactate dehydrogenase (LDH) and glucose were measured in cell‐free supernatant, after samples were centrifuged at 4000 rpm for 20 min (Universal 320, Hettich, Tuttlingen, Germany). Lactate and extracellular K^+^ were analysed by potentiometry using ABL835 (Radiometer, Krefeld, Germany). Measurements for free Hb were done by ultraviolet–visible (UV/VIS) spectroscopy (Specord50/Analytik Jena, Jena, Germany), LDH and glucose by UV/VIS photometry (Cobas 8000, Roche, Basel, Switzerland). To independently verify the K^+^ values, K^+^ concentrations were also measured externally by flame photometry in 12 selected samples of Series C. Adenosine triphosphate (ATP) was analysed using colorimetry by the hexokinase method (Hexokinase UV‐Test, Greiner Diagnostic GmbH, Bahlingen, Germany) according to the manufacturer's instructions. Samples of irradiated RBCs were collected within 2 h after irradiation and within 1 h after washing.

Statistical analyses used the Mini Tab Version 20 (State College, Pennsylvania) and R version 4.2.2 (Free Software Foundation). Mixed‐effects regression analyses evaluated treatment effects across outcomes. Linear mixed models were fitted for each outcome variable, considering fixed effects for treatment and time points and random intercepts for patients accounting for repeated measures. Analyses were restricted to post‐Day‐8 measurements of Series C and fitted using lmer (lme4 package) in R. Paired *t*‐tests with 95% confidence intervals (CIs) were conducted to assess significant differences across the series. The 95% CIs for the mean estimates were calculated based on a *t*‐distribution with *n* − 1 degrees of freedom. For comparisons of individual time points between washed and non‐washed samples of Series C, Bonferroni's approach was applied to correct for multiple testing. Non‐inferiority within the framework of a one‐sided *t*‐test was analysed. We established non‐inferiority using a margin equal to 50% of one standard deviation observed in Day 1 control samples. Non‐inferiority was concluded if the entire 95% CI of the estimated difference fell within the margin boundaries, taking into account the clinically meaningful direction of the effect. Specifically, for measures where lower values indicate improvement, the upper bound of the 95% CI had to be below the positive margin; for measures where higher values indicate improvement, the lower bound of the 95% CI had to be above the negative margin.

## RESULTS

**FIGURE 2:**
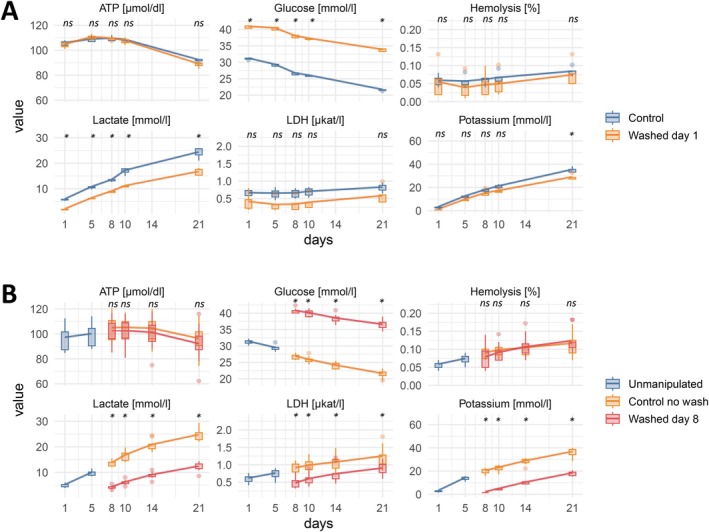
Quality parameters of red blood cells (RBCs) in experimental Series A and B. Splitting and washing were performed as shown in Figure 1. Shown are adenosine triphosphate (ATP) concentration (μmol/dL), glucose levels (mmol/L), haemolysis (%), lactate concentration (mmol/L), LDH activity (μkat/L) and potassium levels (mmol/L). Series A compares control samples with units washed on Day 1; Series B compares unmanipulated samples with unwashed controls and units washed on Day 8. Standard boxplots show the interquartile range (IQR, 25th–75th percentiles), whiskers extend to the most extreme data point within 1.5 × IQR and individual points show outliers beyond this range. Lines connect mean values across time points. Asterisks indicate statistical significance *p* < 0.01; ns, not significant (see also Table 1).

We aimed to characterize the quality of RBCs with a focus on their use in perinatal massive transfusion. The experimental layout is shown in Figure 1. First, we studied the influence of a washing step either on Day 1 after donation (Series A) or on Day 8 after donation (Series B; Figure 2a,b). We found that haemolysis remained in the range of the levels of unwashed controls in both series. Levels of ATP were slightly lower in washed products compared with unwashed controls. LDH and lactate were reduced after washing, and glucose levels increased after washing on either Day 1 or 8 (Figure 2). In contrast, concentrations of K^+^ were lowered by washing on Day 8 much more effectively than by washing on Day 1 (Figure 2a,b). Statistical significance was reached for glucose and lactate and in part for K^+^ for Series A and for glucose, lactate, LDH, K^+^ in part for ATP measurements in Series B (Figure 2, Table 1). Data of extended storage until Day 42 are given in Table S1. Taken together, the washing step on Day 1 had a major influence on residual glucose and lactate, whereas washing on Day 8 in addition resulted in significant alterations of lactate, LDH and K^+^.

**TABLE 1:** Statistical significances Series A and B.

Parameter		1 day	5 days	8 days	10 days	14 days	21 days
Series A							
Glucose (mmol/L)	*p* value	<0.01	<0.01	<0.01	<0.01	n.d.	<0.01
	Bonferroni correction	**<0.01**	**<0.01**	**<0.01**	**<0.01**	n.d.	**<0.01**
Lactate (mmol/L)	*p* value	<0.01	<0.01	<0.01	<0.01	n.d.	0.02
	Bonferroni correction	**<0.01**	**<0.01**	**<0.01**	**0.01**	n.d.	0.08
Haemolysis (%)	*p* value	0.19	0.21	0.39	0.21	n.d.	0.53
	Bonferroni correction	0.77	0.85	1.56	0.85	n.d.	2.12
LDH (μkat/L)	*p* value	0.12	0.01	0.03	0.04	n.d.	0.10
	Bonferroni correction	0.49	0.06	0.1	0.14	n.d.	0.42
Potassium (mmol/L)	*p* value	0.01	<0.01	0.04	<0.01	n.d.	<0.01
	Bonferroni correction	**0.05**	**0.02**	0.17	**0.01**	n.d.	**<0.01**
ATP (mmol/L)	*p* value	0.17	0.12	0.93	0.62	n.d.	0.12
	Bonferroni correction	0.68	0.47	3.71	2.48	n.d.	0.46
Series B							
Glucose (mmol/L)	*p* value	n.a.	n.a.	<0.01	<0.01	<0.01	<0.01
	Bonferroni correction	n.a.	n.a.	**<0.01**	**<0.01**	**<0.01**	**<0.01**
Lactate (mmol/L)	*p* value	n.a.	n.a.	<0.01	<0.01	<0.01	<0.01
	Bonferroni correction	n.a.	n.a.	**<0.01**	**<0.01**	**<0.01**	**<0.01**
Haemolysis (%)	*p* value	n.a.	n.a.	0.3	0.43	0.88	0.57
	Bonferroni correction	n.a.	n.a.	1.21	1.72	3.52	2.3
LDH (μkat/L)	*p* value	n.a.	n.a.	<0.01	<0.01	0.01	<0.01
	Bonferroni correction	n.a.	n.a.	**<0.01**	**<0.01**	**0.03**	**0.02**
Potassium (mmol/L)	*p* value	n.a.	n.a.	<0.01	<0.01	<0.01	<0.01
	Bonferroni correction	n.a.	n.a.	**<0.01**	**<0.01**	**<0.01**	**<0.01**
ATP (mmol/L)	*p* value	n.a.	n.a.	0.01	0.02	0.02	0.01
	Bonferroni correction	n.a.	n.a.	**0.03**	0.09	0.09	**0.02**

*Note*: Pairwise comparisons were conducted using paired *t*‐tests at each time point. To adjust for the number of time points tested, a Bonferroni correction was applied to account for multiple comparisons. Values in bold indicate statistical significance of Bonferroni correction results. Statistical significance was accepted at *p* ≤ 0.05.

Abbreviations: ATP, adenosine triphosphate; LDH, lactate dehydrogenase; n.a., not applicable.

In a third series of experiments (C), we analysed the influence of irradiation on RBC quality, with and without washing. To model a ‘worst‐case’ scenario for use of RBCs in a neonatal setting, we chose to manipulate the units on Day 8 of storage, which is a maximum recommended age for RBCs used for massive transfusion in small children. Any units transfused at earlier time points are expected to display a less pronounced storage lesion.

**FIGURE 3:**
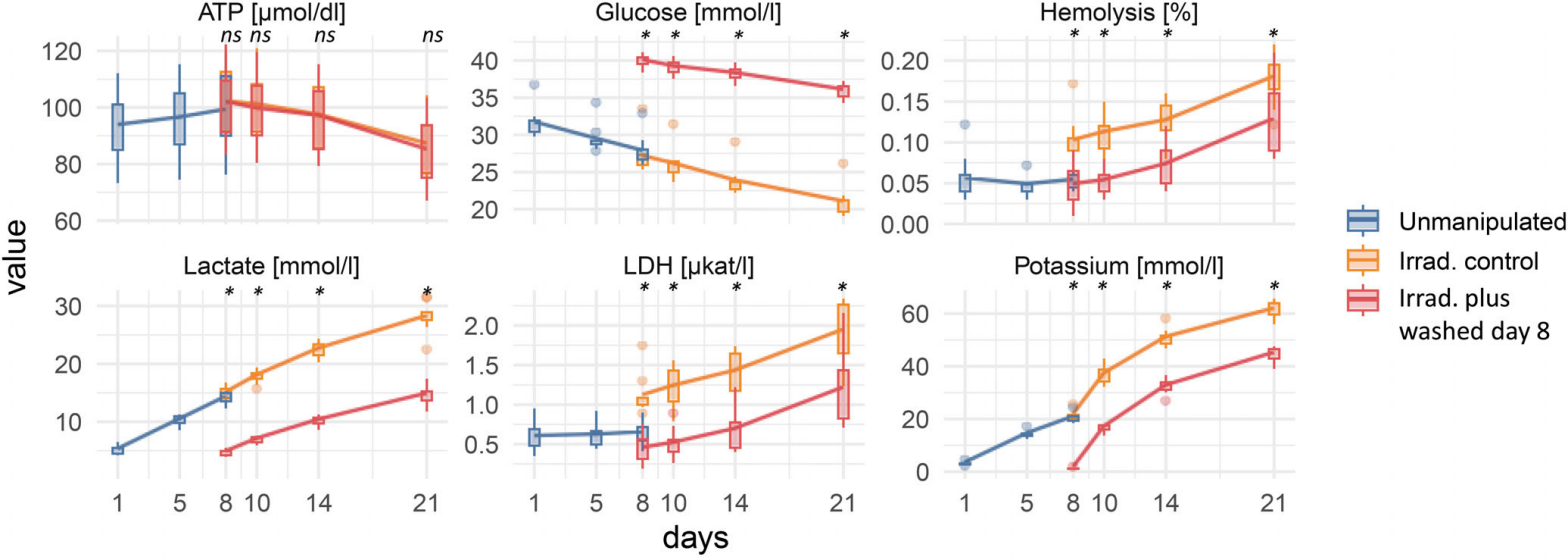
Quality parameters of red blood cells (RBCs) in experimental Series C. Splitting, irradiation and washing were performed as shown in Figure 1. Shown are adenosine triphosphate (ATP) concentration (μmol/dL), glucose levels (mmol/L), haemolysis (%), lactate concentration (mmol/L), LDH activity (μkat/L) and potassium levels (mmol/L). The series compares unmanipulated samples with irradiated controls and units washed on Day 8. Standard boxplots show the interquartile range (IQR; 25th–75th percentiles), whiskers extend to the most extreme data point within 1.5 × IQR and individual points show outliers beyond this range. Lines connect mean values across time points. Asterisks indicate statistical significance *p* < 0.01; ns, not significant (see also Table 2).

First, we analysed a time series of irradiated washed versus irradiated unwashed samples to determine whether the washing process itself had an effect on the quality parameters (Figure 3). Using a linear mixed model analysis that accounted for repeated measures, we found significant differences between irradiated washed versus irradiated unwashed samples for all characteristics except ATP (*p* < 0.05; Table S2). For all parameters, we conducted pairwise comparisons using paired *t*‐tests at each time point. To account for the number of time points tested, we applied a Bonferroni correction for multiple comparisons (Table 2). Except for ATP, all analysed parameters showed statistically significant differences throughout the observation period. When comparing the Day 8‐irradiated and non‐irradiated controls, which represent comparison between different RBCs (not from the same splits), a >50% increase in haemolysis was seen in the irradiated RBCs and in K^+^ already on Day 10 (36.8 ± 2.2 mM irradiated vs. 22.5 ± 1.6 mM in controls). While there was an increase, haemolysis met acceptance criteria for all units (<1% [United States], <0.8% [European Union]).

**TABLE 2:** Statistical significance Series C.

Parameter		8 days	10 days	14 days	21 days
Glucose (mmol/L)	Effect	12.91	13.06	14.44	15.02
	95% CI	(14.13; 11.68)	(11.59; 14.53)	(13.36; 15.51)	(13.81; 16.24)
	*p* value	<0.01	<0.01	<0.01	<0.01
	*p* value Bonferroni correction	**<0.01**	**<0.01**	**<0.01**	**<0.01**
Lactate (mmol/L)	Effect	−10.27	−11.00	−12.30	−13.43
	95% CI	(−10.26; −9.61)	(−11.67; −10.31)	(−13.18; −11.42)	(−15.31; −11.55)
	*p* value	<0.01	<0.01	<0.01	<0.01
	*p* value Bonferroni correction	**<0.01**	**<0.01**	**<0.01**	**<0.01**
Haemolysis (%)	Effect	−0.05	−0.06	−0.05	−0.05
	95% CI	(−0.07; −0.04)	(−0.08; −0.04)	(−0.07; −0.04)	(−0.08; −0.03)
	*p* value	<0.01	<0.01	<0.01	<0.01
	*p* value Bonferroni correction	**<0.01**	**<0.01**	**<0.01**	**<0.01**
LDH (μkat/L)	Effect	−0.67	−0.72	−0.68	−0.73
	95% CI	(−0.79; 0.55)	(−0.90; −0.54)	(−0.90; −0.45)	(−1.00; −0.46)
	*p* value	<0.01	<0.01	<0.01	<0.01
	*p* value Bonferroni correction	**<0.01**	**<0.01**	**<0.01**	**<0.01**
Potassium (mmol/L)	Effect	−20.08	−20.22	−18.36	−16.66
	95% CI	(−21.14; −19.02)	(−22.10; −18.34)	(−20.54; −16.18)	(−19.37; −13.94)
	*p* value	<0.01	<0.01	<0.01	<0.01
	*p* value Bonferroni correction	**<0.01**	**<0.01**	**<0.01**	**<0.01**
ATP (mmol/L)	Effect	−0.29	−1.41	−0.35	−2.03
	95% CI	(−2.73; 2.15)	(−4.03; 1.21)	(−3.22; 2.52)	(−5.13; 1.08)
	*p* value	0.80	0.26	0.80	0.176
	*p* value Bonferroni correction	3.18	1.04	3.18	0.7

*Note*: Pairwise comparisons were conducted using paired *t*‐tests at each time point. To adjust for the number of time points tested, a Bonferroni correction was applied to account for multiple comparisons. Values in bold indicate statistical significance of Bonferroni correction results. Statistical significance was accepted at *p* value ≤0.05.

Abbreviations: ATP, adenosine triphosphate; CI, confidence interval; LDH, lactate dehydrogenase.

The additional washing step in Day 8‐irradiated RBCs resulted in only minor alterations of ATP levels during subsequent storage compared to the unwashed controls (Figure 3). Glucose levels were found to be increased between 1.5‐fold and 2‐fold after irradiation plus an additional washing step, compared with irradiated non‐washed products over the storage time, whereas LDH and lactate concentrations were found to be about half compared to the non‐washed irradiated controls, which is in line with ongoing red cell metabolism in the first days after separation. A significantly lower haemolysis rate was evident in washed irradiated RBCs compared to non‐washed irradiated products (Figure 3, Table 2).

Washing after irradiation on Day 8 reduced K^+^ concentration to 1.12 ± 0.05 mM versus 21.20 ± 2.78 mM in unwashed controls (means ±95% CI; Figure 3 and Table S1). Two days later, on Day 10, K^+^ levels in the washed units increased to 16.58 ± 0.85 mM. The effect of washing on K^+^ levels is highly significant across all time points examined (*p* < 0.01; Table 2). In the further time course, K^+^ levels approached up to 0.8‐fold of the levels of unwashed irradiated units, which is relatively faster than in Series B which were washed but not irradiated (Figures 2 and 3).

In 12 selected samples of Series C, K^+^ was independently determined also by flame photometry. The mean values deviated by 3.4% from the value determined by ABL835 controls measured using potentiometry (Table S3).

Furthermore, all endpoints of RBCs irradiated and washed on Day 8 were non‐inferior to the unmanipulated samples from Day 1 using a non‐inferiority margin of 50% of the standard deviation observed between samples from the unmanipulated Day 1 samples (*p* < 0.025, Table S4). We also analysed a potential effect of irradiation by analysing samples immediately before and immediately after irradiation, which did not show significant differences (Table S5).

Taken together, washing led to elevated glucose and lower lactate and LDH values but influenced haemolysis only after irradiation. Our data show that irradiation has a negative effect on K^+^ concentrations in RBCs upon storage. Unmanipulated RBCs stored in SAGM showed K^+^ levels below 15 mM until Day 5 and below 25 mM until Day 8, including the 95% CI. Washing of Day 8‐irradiated RBCs was able to keep concentrations of K^+^ below 25 mM for 2 more days.

## DISCUSSION

We validated essential quality parameters in three series of washed and/or irradiated split RBCs. Thus, K^+^ load per transfusion can be controlled for routine production of RBCs, for example, for use in high‐volume RBC transfusions in trauma or cardiac surgery with EC, where posttransfusion levels of K^+^ ranging from 6.3 to 12 mM in conjunction with TAHCA have been described [1, 4, 15, 16].

Recommendations for pre‐ and perinatal transfusions state that RBCs should be irradiated only shortly before transfusion, for example, within 12–24 h [10, 17]. We determined K^+^ values in RBCs after irradiation on Day 8 and further storage. Our data show that K^+^ values can increase from 21.2 ± 1.0 mM on Day 8 to 36.8 ± 2.1 mM (means ± SD) within another 2 days. But K^+^ levels as low as 19.9 and 30 mM in blood components have also been associated with TAHCA [4, 5]. Previous work has also reported K^+^ concentrations of 45 ± 3 mM on Day 11 in RBCs irradiated on Day 7 and of 62 ± 11 mM at the end of storage time (Day 42) [18, 19].

The data by Olafson et al. from small units [20] and of Marks et al. [13] looking at normal‐sized 1‐week‐old RBC units yielded lower concentrations of K^+^ (15 mM) than our study (20 mM) or that of Grabmer et al. [21] who analysed RBC collected by apheresis (30 mM). This shows that K^+^ concentrations in 7‐day‐old units vary with different production schemes and that K^+^ values between 15 and 30 mM can be expected in RBCs after 1 week of storage. These results provide a rationale to individually validate K^+^ concentrations in paediatric RBC units in blood centres that deliver to paediatric intensive care units and to provide a defined RBC quality to transfusing paediatricians.

Currently, there are two main indications to wash RBCs. One is to reduce allergic reactions, and the other is to reduce extracellular potassium in the supernatant [7, 8, 9, 10]. In our hands, washing of RBC units on either Day 1 or 8 resulted in 25%–50% improved glucose, lactate and LDH levels during subsequent storage, whereas ATP levels and haemolysis were only slightly influenced. Our haemolysis data conflict somewhat with others who found a 2‐fold increase in haemolysis after washing [12, 22]. This may be explained by the fact that, in contrast to our study, these authors did not centrifuge the unwashed controls. Like Loh et al. [12], we found lower lactate and K^+^ concentrations after washing than in the controls.

Irradiation on Day 8 produced an approximately 2‐fold increase of K^+^ within 2 days of further storage. Previous work has found K^+^ concentrations to rise at similar rates after washing [11, 13, 14]. Loh et al. found an advantage of washing in its effective reduction of biological response modifiers such as proinflammatory cytokines [12]. Re‐addition of SAGM after washing of RBCs resulted in increased glucose concentrations compared with controls. However, this was not associated with increased ATP, indicating that glycolysis in the RBCs was unaffected by the washing step.

Limitations of our study include the possibility of inherent factors inducing a more rapid decay of stored erythrocytes such as glucose‐6‐phosphate dehydrogenase deficiency. Furthermore, the volumes studied here are not aligned with paediatric RBC volumes after dividing units into four. Also, our study did not include blood bag systems provided by different manufacturers. One can wash red cells by centrifugation with closed tube connections, as described in our study, or by an open system, for example, a COBE2991. In the latter case, a 24 h time limit of further storage is imposed for safety, whereas a closed system, as in our study, allows storage for 2 more days after washing.

In summary, the production of RBCs with limited K^+^ concentrations and reproducible product quality regarding ATP, haemolysis and lactate is feasible. They can reduce transfusion‐associated hyperkalaemic events in premature and critically ill infants requiring massive RBC transfusion. Our data show that RBCs stored for 8 days are safe, and when washed, can be used for up to 2 more days. Blood services should validate and regularly control parameters for massive transfusions in preterm infants and critically ill children. In our own routine practice, we can provide an RBC pool for paediatric transfusion, limited by further selection criteria for neonatal transfusions such as cytomegalovirus (CMV) status, O Rh(D) negative type or negativity for other antigens. This can be achieved with an RBC depot of 1000 units with an average RBC turnaround time of about 1.5 weeks. Around 10 suitable fresh (up to 8 days old), CMV negative, O Rh(D) negative RBC units can be available for neonatal transfusions with our blood donor pool. We thus recommend washing with and without irradiation on Day 8 at the latest for the production of paediatric RBCs as a strategy to reduce extracellular K^+^ levels. This way, additional storage of up to 2 more days can be allowed.

## CONFLICT OF INTEREST STATEMENT

The authors declare no conflicts of interest.

## Supporting information


**Table S1.** Quality parameters in RBCs over storage time. Values are means ± 95% confidence interval; *n* = 4 (Series A) or *n* = 11 (Series B and C). irrad., irradiated; n.d., not determined. *Statistical significance was accepted at *p*‐values less or equal to 0.05. For statistical significances please refer to Figures 2 and 3, and Tables 1 and 2.

**Table S2.** Mixed model analysis in experimental Series C. Calculations were performed as described in materials and methods.

**Table S3.** Comparison of K^+^ measurements with two methods. Twelve selected samples were in addition analysed by flame photometry. The mean value using potentiometry was 0.8% higher that with flame photometry; *p* = 0.9 (non‐significant) using two‐sided *t* test.

**Table S4.** Noninferiority analysis in experimental Series C. Calculations were performed as described in materials and methods and the results section.

**Table S5.** Influence of irradiation in experimental Series C. Values of the experiment in Series C were analysed for influence of the irradiation immediately before and after the irradiation using a non‐inferiority test as described in materials and methods.

## Data Availability

The data that support the findings of this study are available from the corresponding author upon reasonable request.

## References

[1] Lee AC , Reduque LL , Luban NL , Ness PM , Anton B , Heitmiller ES . Transfusion‐associated hyperkalemic cardiac arrest in pediatric patients receiving massive transfusion. Transfusion. 2014;54:244–254. 23581425 10.1111/trf.12192

[2] OReilly MF , Bruno CD , Prudencio TM , Ciccarelli N , Guerrelli D , Nair R , et al. Potential consequences of the red blood cell storage lesion on cardiac electrophysiology. J Am Heart Assoc. 2020;9:e017748. 33086931 10.1161/JAHA.120.017748 PMC7763412

[3] Ayach T , Nappo RW , Paugh‐Miller JL , Ross EA . Postoperative hyperkalemia. Eur J Intern Med. 2015;26:106–111. 25698564 10.1016/j.ejim.2015.01.010

[4] Burke M , Sinha P , Luban NLC , Posnack NG . Transfusion‐associated hyperkalemic cardiac arrest in neonatal, infant, and pediatric patients. Front Pediatr. 2021;9:765306. 34778153 10.3389/fped.2021.765306 PMC8586075

[5] Vraets A , Lin Y , Callum JL . Transfusion‐associated hyperkalemia. Transfus Med Rev. 2011;25:184–196. 21498041 10.1016/j.tmrv.2011.01.006

[6] Fergusson DA , Hébert P , Hogan DL , LeBel L , Rouvinez‐Bouali N , Smyth JA , et al. Effect of fresh red blood cell transfusions on clinical outcomes in premature, very low‐birth‐weight infants: the ARIPI randomized trial. JAMA. 2012;308:1443–1451. 23045213 10.1001/2012.jama.11953

[7] Guide to the preparation, use and quality assurance of blood components: Recommendation no. R(95) 15. 20th ed. Strasbourg: European Directorate for the Quality of Medicines & HealthCare; 2020.

[8] New HV , Berryman J , Bolton‐Maggs PHB , Cantwell C , Chalmers EA , Davies T , et al. Guidelines on transfusion for fetuses, neonates and older children. Br J Haematol. 2016;175:784–828. 27861734 10.1111/bjh.14233

[9] Cohn C , Delaney M , Johnson ST , Katz LM , Schwartz J , editors. Technical Manual, 21st ed. American Association of Blood Banks. Available from: https://www.aabb.org/aabb-store/product/technical-manual-21st-edition---digital-16919069. Last accessed 11 Feb 2025.

[10] Recommandation de bonne pratique – Transfusion de globules rouges homologues – Produits, indications, alternatives: Méthode Recommendatinos pour la pratique clinique. Haute Autorité de santé, Republique Francaise 2015. Available from: https://www.has‐sante.fr/upload/docs/application/pdf/2015‐02/transfusion_de_globules_rouges_homologues_‐_produits_indications_alternatives_‐_recommandations.pdf. Last accessed 11 Feb 2025.

[11] Weiskopf RB , Schnapp S , Rouine‐Rapp K , Bostrom A , Toy P . Extracellular potassium concentrations in red blood cell suspensions after irradiation and washing. Transfusion. 2005;45:1295–1301. 16078915 10.1111/j.1537-2995.2005.00220.x

[12] Loh YS , Tan S , Kwok M , Stark MJ , Marks DC . Reduction of biological response modifiers in the supernatant of washed paediatric red blood cells. Vox Sang. 2016;111:365–373. 27864978 10.1111/vox.12442

[13] Marks DC , Webb RG , Linnane C , Aung HH , Dennington PM , Tan JCG . X‐ and gamma‐irradiation have similar effects on the in vitro quality of stored red cell components. Transfusion. 2021;61:3214–3223. 34510450 10.1111/trf.16656

[14] Shih AW , Apelseth TO , Cardigan R , Marks DC , Bégué S , Greinacher A , et al. Not all red cell concentrate units are equivalent: international survey of processing and in vitro quality data. Vox Sang. 2019;114:783–794. 31637738 10.1111/vox.12836

[15] Raza S , Ali Baig M , Chang C , Dabas R , Akhtar M , Khan A , et al. A prospective study on red blood cell transfusion related hyperkalemia in critically ill patients. J Clin Med Res. 2015;7:417–421. 25883703 10.14740/jocmr2123w PMC4394913

[16] Livingston MH , Singh S , Merritt NH . Massive transfusion in paediatric and adolescent trauma patients: incidence, patient profile, and outcomes prior to a massive transfusion protocol. Injury. 2014;45:1301–1306. 24950797 10.1016/j.injury.2014.05.033

[17] Reeves HM , Goodhue Meyer E , Harm SK , Lieberman L , Pyles R , Rajbhandary S , et al. Neonatal and pediatric blood bank practice in the United States: results from the AABB pediatric transfusion medicine subsection survey. Transfusion. 2021;61:2265–2276. 34110629 10.1111/trf.16520

[18] Serrano K , Chen D , Hansen AL , Levin E , Turner TR , Kurach JDR , et al. The effect of timing of gamma‐irradiation on hemolysis and potassium release in leukoreduced red cell concentrates stored in SAGM. Vox Sang. 2014;106:379–381. 24330144 10.1111/vox.12112

[19] de Korte D , Thibault L , Handke W , Harm SK , Morrison A , Fitzpatrick A , et al. Timing of gamma irradiation and blood donor sex influences in vitro characteristics of red blood cells. Transfusion. 2018;58:917–926. 29341199 10.1111/trf.14481

[20] Olafson C , William N , Howell A , Beaudin L , Gill B , Clarke G , et al. Preparing small‐dose red cell concentrates (RCCs) for neonatal and pediatric transfusions: impact of RCC volume, storage, and irradiation. Transfusion. 2022;62:1506–1510. 35869790 10.1111/trf.17027

[21] Grabmer C , Holmberg J , Popovsky M , Amann E , Schönitzer D , Falaize S , et al. Up to 21‐day banked red blood cells collected by apheresis and stored for 14 days after automated wash at different times of storage. Vox Sang. 2006;90:40–44. 16359354 10.1111/j.1423-0410.2005.00719.x

[22] O'Leary MF , Szklarski P , Klein TM , Young PP . Hemolysis of red blood cells after cell washing with different automated technologies: clinical implications in a neonatal cardiac surgery population. Transfusion. 2011;51:955–960. 21091957 10.1111/j.1537-2995.2010.02935.x

